# Validating the AIM–N: An AI-motivation and needs scale with multi-group invariance and MIMIC-DIF evidence in higher education

**DOI:** 10.1371/journal.pone.0341134

**Published:** 2026-03-13

**Authors:** Laura Maska, Patra Vlachopanou, Dimitrios Kalamaras, Angeliki Tsameti

**Affiliations:** Aegean College, Greece; University of Tartu, ESTONIA

## Abstract

The rapid adoption of generative AI in higher education raises critical questions about its impact on student motivation and basic psychological needs. This study introduces and validates the AI-Motivation and Needs (AIM-N) scale, a new instrument assessing how AI integration influences students’ motivational orientations and need satisfaction in learning. Survey data were collected from N = 904 university students. A confirmatory factor analysis (CFA) supported a multi-factor structure for the AIM-N, comprising two subscales of AI-related redundancy beliefs (task-level and motivational-level) and three subscales of AI-related motivational orientations (intrinsic, identified, controlled), with acceptable model fit (CFI ≈ 0.96, TLI ≈ 0.95, RMSEA ≈ 0.05) and strong factor loadings. Internal consistency was good for most subscales (Cronbach’s α = 0.70–0.90; McDonald’s ω in similar range), except a single-item amotivation indicator. Multi-group CFA indicated that the AIM-N achieved configural, metric, and scalar invariance across gender, study level (Bachelor’s, Master’s, PhD), academic field, and frequency of AI use (ΔCFI < 0.01), after minor modifications for the AI-use groups. A MIMIC model (Multiple Indicators, Multiple Causes) revealed that higher AI tool usage was associated with stronger beliefs that AI renders learning tasks redundant and slightly more controlled motivation (β ≈ 0.30 and 0.21, p <.001), while gender showed no significant effects. Field of study had significant impacts: STEM students reported higher redundancy beliefs and controlled motivation than humanities students (p <.01). The MIMIC analysis also identified differential item functioning (DIF) for certain items; for example, students in competitive fields endorsed the “pressure to use AI” item more than expected from their latent trait levels. These results demonstrate that the AIM-N is a reliable and valid instrument for measuring the nuanced ways AI influences student motivation and needs. The discussion addresses theoretical implications for Self-Determination Theory in the age of AI, practical implications for educators, and recommendations for future research on sustaining meaningful student engagement when AI tools are pervasive.

## Introduction

The rapid spread of generative AI in higher education has intensified debate about its effects on students’ motivation and the meaning they ascribe to learning. On the upside, AI assistants—such as large language models—can act as scalable, adaptive supports, continuing a long tradition of AI-powered tutoring systems that have shown moderate learning gains [1] and promising early results in hybrid human-AI tutoring scenarios [2]. At the same time, educators have expressed concern that the effort-saving affordances of generative AI may prompt automation bias and cognitive offloading, leading to shallower learning and diminished personal investment [3,4]. Recent systematic reviews and global surveys suggest that students frequently use AI tools such as ChatGPT for brainstorming, summarization, and even full assignment completion—raising concerns about academic integrity, reliability, and a creeping sense of redundancy [5,6]. From a motivational perspective, these dynamics raise an urgent question: if an AI can effortlessly produce a well-formed essay or solve a problem set, what is the perceived value of investing one’s own time and effort? Emerging anecdotal and theoretical accounts suggest that some students increasingly view coursework as pointless or merely procedural [7,8], potentially threatening the fulfillment of autonomy, competence, and relatedness that undergird intrinsic motivation and academic purpose [9]. To move beyond speculation, there is a pressing need for empirical work that captures how AI availability intersects with students’ motivational beliefs and basic psychological needs in authentic learning contexts across disciplines.

Self-Determination Theory (SDT) offers a robust framework for explaining students’ motivation and basic psychological needs in technology-rich learning contexts, including the growing presence of AI in coursework [10,11]. SDT differentiates intrinsic motivation (engaging out of interest and enjoyment) from qualitatively different forms of extrinsic motivation that vary in the degree of internalization—from external regulation (behavior driven by rewards or pressures) and introjected regulation (behavior to avoid guilt or obtain approval), to more autonomous forms such as identified regulation (valuing and personally endorsing the goal) and, at the far end, integrated regulation (alignment with one’s sense of self) [10,12]. SDT also recognizes amotivation, in which the activity lacks perceived value or intentionality [12]. Central to this framework is the proposition that satisfying the three basic psychological needs—autonomy, competence, and relatedness—supports optimal motivation, engagement, and well-being in learning [13], a claim repeatedly corroborated in classroom research [14,15].

The introduction of AI tools into students’ academic work plausibly modulates these motivational dynamics. Autonomy may be undermined if students perceive social or instructional pressure to use AI in particular ways (promoting more controlled forms of regulation), yet enhanced when AI affords choice and self-direction in strategy and pacing [15,13]. Competence may be strengthened when AI scaffolds success and feedback, but it may also erode if heavy reliance supports self-doubt or cognitive offloading that reduces opportunities to experience mastery [3,13]. Effects on relatedness are likely indirect—emerging as AI reshapes collaborative practices, instructor–student interaction, and norms of co-creation in coursework [13,16]. From an SDT perspective, therefore, the key empirical question is how AI availability shifts the quality of motivation (from controlled to autonomous), and how it affects need satisfaction across disciplines and task types.

Empirical research on how AI affects student motivation is still nascent, but early findings are instructive. In a large, multi-university network analysis of students learning AI (as subject matter), introjected regulation emerged as the most central node in the motivational system, while intrinsic motivation showed relatively low centrality—suggesting that many students engage with AI more from obligation or social pressure than enjoyment [17]. This pattern contrasts with educators’ aspirations that students employ AI in self-determined ways that support curiosity, volition, and competence rather than as a shortcut that undermines personal growth [9]. Concurrently, instrument work tailored to AI is only beginning to appear—for example, a recent AI Motivation Scale grounded in self-determination theory—emphasising both the momentum and the measurement gap in this domain [18]. To study these issues rigorously, valid measures are needed that capture AI-specific experiences alongside established constructs. Classic instruments such as the Academic Motivation Scale [19] and the Basic Psychological Need Satisfaction and Frustration Scale [20] are widely used and theoretically aligned with SDT, but they do not explicitly address AI. Adding AI-contextualized items—for example, “Using AI makes much of my coursework feel like a formality”—should capture unique variance in motivational beliefs and need satisfaction/frustration that standard scales may miss [7].

The present study introduces and validates the AI-Motivation and Needs (AIM–N) scale, which appends AI-related extensions to established SDT-based motivation and needs measures for higher education. Accordingly, the present study followed a standard multi-step scale validation procedure, including exploratory and confirmatory factor analyses conducted on independent subsamples, tests of measurement invariance across key groups, and complementary DIF analyses. AIM–N is designed to assess: (a) students’ beliefs that AI renders specific learning tasks—or learning itself—redundant or less meaningful, and (b) how AI usage relates to their motivational orientations (intrinsic, identified, controlled, amotivation) and need satisfaction/frustration (with emphasis on autonomy and competence). A comprehensive psychometric evaluation of the AIM–N was conducted using data from a large sample of higher education students, with analyses focused on construct validity, measurement invariance, and item-level functioning. Specifically, we: (1) test the factor structure via CFA (and compare with alternative structures); (2) estimate reliability (α, ω) and construct validity (convergent, discriminant, and criterion validity with AI use); (3) examine measurement invariance across gender, field (STEM vs. non-STEM), and study level (UG vs. PG); (4) implement MIMIC-DIF to detect potential item bias by covariates; and (5) evaluate incremental validity beyond AMS/BPNSFS in predicting AI-related academic outcomes (e.g., perceived task value, engagement).

Specifically, the following objectives were addressed:

- **Confirmatory Factor Analysis (CFA):** Test the factor structure of the AIM-N items, expecting a multi-factor model corresponding to theorized subscales (e.g., Redundancy Beliefs, AI-Related Intrinsic Motivation, etc.). Model fit and internal consistency for each subscale were evaluated using Cronbach’s α and McDonald’s ω coefficients.
- **Measurement Invariance:** Examine whether the AIM-N measures constructs equivalently across key groups: gender (male, female), level of study (undergraduate, postgraduate, doctoral), field of study, and self-reported AI usage frequency. Support for configural, metric, and scalar invariance would indicate the scale functions uniformly across these groups.
- **MIMIC and DIF Analysis:** Use a Multiple-Indicator Multiple-Cause structural model to assess how external variables (AI usage frequency, gender, field of study, etc.) predict the latent factors, and to detect any Differential Item Functioning (DIF) – specific item biases – by including direct paths from covariates to item indicators. This allows identifying if certain item responses are influenced by group membership beyond the latent trait.

Establishing the reliability and validity of the AIM–N provides a timely, psychometrically defensible instrument for interrogating students’ motivational experiences in the age of AI. In accordance with contemporary testing standards, multiple sources of validity evidence were gathered to support the use of the AIM–N instrument (internal structure, relations to other variables, and item functioning) and report reliability with appropriate coefficients (e.g., ω), aligning with the Standards for Educational and Psychological Testing and best-practice recommendations for scale evaluation [21,22]. Because fair score interpretation depends on equivalence, measurement invariance was examined across key groups relevant to higher education (e.g., gender, study level, and STEM vs. non-STEM fields) and complement multi-group CFA with MIMIC-DIF to detect potential item bias [23–24].

Substantively, AIM–N enables educators and researchers to examine whether AI integration is associated with diminished task value and perceived redundancy—or whether students sustain self-determined engagement when instruction supports basic psychological needs. This question is central to expectancy–value theory (task value) and self-determination theory (autonomy, competence, relatedness) [9,25,26]. If AI makes routine coursework feel procedural, task value may erode; conversely, when courses foreground autonomy-supportive design, authentic problem-solving, and competence-building with AI as a tool rather than a crutch, students’ high-quality motivation can be sustained [7,14,27].

For policy and practice, validated scores from AIM–N can guide targeted interventions—e.g., redesigning assessments for authenticity, emphasizing metacognition and process, and adopting AI-literate, autonomy-supportive pedagogy in science and technology courses—while monitoring equity via invariance and DIF diagnostics. Such actions align with emerging guidance on responsible AI in education that balances innovation with human agency and meaningful learning [28,29]. In short, AIM–N offers a robust lens for diagnosing where AI may threaten perceived meaning and where instructional design can protect or enhance students’ motivation in AI-rich higher education.

### Study aims and research questions

This study introduces and validates the AI-Motivation and Needs (AIM–N) instrument for higher education. Psychometric objectives were to (a) confirm the internal structure via CFA, (b) establish reliability (α, ω), (c) test measurement invariance across gender, study level, academic field, and AI-use frequency, and (d) examine MIMIC-DIF to evaluate item bias and covariate effects. Substantively, the study asked:

RQ1. How do AI-specific redundancy beliefs and AI-related motivational orientations (intrinsic, identified, controlled) relate at the latent level?

RQ2. Do AIM–N measures function equivalently across key student groups (gender, level, field, AI-use), enabling fair score comparisons?

RQ3. Which student characteristics (AI-use frequency, field) predict higher redundancy beliefs and controlled regulation?

RQ4. Which items exhibit DIF (e.g., “competitive pressure”, “degree losing value”), and how should such items be interpreted in practice?

## Method

### Ethics

Ethics approval and consent. The study protocol (ID OR25AOC2702) was reviewed and approved by the Institutional Research Ethics Committee, in accordance with institutional and national research ethics standards. All procedures adhered to the Declaration of Helsinki and local data-protection regulations. Electronic informed consent was obtained from all participants prior to data collection.The manuscript contains no identifiable personal data or images.

### Participants

Participants were 904 students (64% female, 26% male, 2% no response) enrolled in higher education institutions. Ages ranged from 18 to 54+ (modal 18–24; 45% were 18–24, 25% were 25–34, 16% were 35–44, 11% were 45–54, and ~3% above 54). The mean age was approximately 30 years (reflecting a subset of mature postgraduate students). In terms of study level, 63% were pursuing a Bachelor’s degree, 30% a Master’s, and 4% a doctoral degree, with the remainder in other programs. Academic fields were diverse: the largest groups were Psychology (23%), Health Sciences (20%), Education (15%), Business (12%), and Computer Science (8%), with smaller representation from Engineering, Humanities, Social Sciences, Law, and other fields. The sample thus covered a broad cross-section of disciplines, allowing for invariance testing across field of study. Participants were drawn from multiple universities (primarily in Greece, where the survey was distributed in Greek and English) using convenience and snowball sampling. Participation was voluntary and anonymous.

### Procedure

Data were collected online between the end of April 2025 and the end of June 2025. After reading an information sheet describing the purpose of the study, their rights, and data handling procedures, respondents provided **written informed consent electronically** before beginning the survey. They then completed demographic questions and a battery of questionnaires on motivation, needs, and AI usage.

To encourage honest reporting, participants were informed that their responses would remain confidential, anonymous, and would have no effect on their course outcomes. Only students aged 18 and above were eligible to participate, so no parental or guardian consent was required. The Institutional Research Ethics Committee reviewed the study design and confirmed that full informed consent was required and obtained from all participants.

The survey took approximately 15–20 minutes to complete. As an inclusion criterion, participants needed to be currently enrolled students in higher education (undergraduate or above). No incentives were provided apart from an explanation of the study’s importance.

## Measures

### Terminology

“Generative AI” denotes model-based systems that produce text/code/media from prompts (e.g., large language models). Redundancy beliefs refer to perceptions that AI renders coursework or skills pointless or devalued (task- vs. motivational/identity-level). AI-related motivational orientations follow Self-Determination Theory (intrinsic, identified, controlled). MIMIC-DIF indicates direct effects from observed covariates to item indicators beyond latent factors, used here to flag potential item bias while retaining a compact model.

AI-Motivation and Needs (AIM-N) Scale: The AIM-N instrument consists of items developed to capture AI-specific influences on students’ motivation and basic psychological needs in learning. It was composed by augmenting established scales with novel items referencing AI use. Table 4 (see Results) lists all AIM-N items, their subscale assignments, and factor loadings. Key components include:

**Table 4:** Model fit for measurement invariance tests.

Grouping Variable	Model	χ²(df)	χ²/df	CFI	RMSEA (90% CI)	ΔCFI vs. prev.	Invariance Conclusion
**Gender (M/F)**	Configural	411.2 (250)	1,64	0.963	0.049 (0.041–0.057)	–	Configural ok
	Metric Λ equal	428.7 (264)	1,62	0.961	0.048 (0.040–0.056)	–0.002	Metric invariant (ΔCFI < .01)
	Scalar Λ,τ equal	456.5 (278)	1,64	0.957	0.051 (0.043–0.058)	–0.004	Scalar invariant (ΔCFI < .01)
**Study Level**	Configural (UG/PG)	424.8 (250)	1,69	0.959	0.052 (0.044–0.060)	–	Configural ok
(UG vs. PG/PhD)	Metric	439.3 (264)	1,66	0.956	0.051 (0.043–0.059)	–0.003	Metric invariant
	Scalar	490.7 (278)	1,76	0.945	0.057 (0.050–0.065)	–0.011	*Partial* Scalar (freed item#8)
	Scalar (partial)^a^	472.9 (277)	1,70	0.953	0.053 (0.046–0.061)	–0.003	Scalar invariant after freeing
**Field of Study**	Configural (5 grps)	812.5 (625)	1,3	0.941	0.054 (0.044–0.063)	–	Configural ok
(5 category groups)	Metric	848.3 (665)	1,26	0.935	0.052 (0.043–0.061)	–0.006	Metric invariant
	Scalar	934.0 (705)	1,324	0.915	0.058 (0.050–0.066)	–0.020	*Partial* Scalar (freed 2 items)
	Scalar (partial)^b^	884.5 (703)	1,258	0.927	0.054 (0.046–0.062)	–0.008	Scalar invariant after freeing
**AI Use Frequency**	Configural (5 grps)	845.9 (625)	1,35	0.952	0.052 (0.041–0.061)	–	Configural ok
(Never–Very Often)	Metric	882.4 (665)	1,32	0.947	0.050 (0.040–0.059)	–0.005	Metric invariant
	Scalar	976.6 (705)	1,38	0.929	0.055 (0.046–0.064)	–0.018	*Partial* Scalar (freed 2 items)
	Scalar (partial)^c^	929.3 (703)	1,32	0.938	0.051 (0.042–0.060)	–0.009	Scalar invariant after freeing

**Note.** ^a^Freed intercept for Redundancy item “degree losing value” (higher for UG).

^b^Freed intercepts for Redundancy “degree losing value” (higher in Tech) and Controlled “stay competitive” (higher in Business).

^c^Freed intercepts for Autonomy-satisfaction AI item (Never group) and one Redundancy item (Never group). χ²/df: Acceptable range < 3

- **Redundancy Beliefs Subscale:** Ten custom items on a 5-point Likert scale (1 = Strongly Disagree to 5 = Strongly Agree) assessing the extent to which students perceive academic tasks or skills as unnecessary or devalued due to AI. Example items include: “I sometimes feel that my academic tasks are pointless because AI can complete them better or faster” and “AI tools have made some of my learning experiences feel redundant or outdated.” Item content was informed by emerging student reports of AI-related redundancy and mapped to established motivational frameworks. In Self-Determination Theory, such beliefs align with amotivation and reductions in perceived autonomy/competence [9,19], while in expectancy–value theory they reflect diminished subjective task value (e.g., utility/attainment value) and reduced perceived importance of learning activities [26]. Guided by best practices in scale development and factor-analytic item banking, an optional two-factor representation within the 10 items was considered based on preliminary exploratory analyses.

**Task-Level Redundancy** (items 1–5; focused on specific coursework/tasks) and Motivational/Identity-Level Redundancy (items 6–10; focused on the broader value of one’s degree or skills). This distinction is theoretically coherent with separable task-specific appraisals versus broader value/identity concerns and is consistent with recommendations for evaluating multidimensional structures during instrument development [26,30,31].

Scoring/interpretation. Higher scores indicate stronger endorsement of the belief that “learning no longer matters” because AI can perform the work, consistent with elevated amotivation and lower task value in established motivational models [9,19,26].

- **The AI-Related Motivational Orientations Subscale** comprised 7 items on a 7-point Likert scale (1 = “Does not correspond at all” to 7 = “Corresponds exactly”) designed to assess how AI availability shapes students’ reasons for engaging in learning. Items were added to an adapted Academic Motivation Scale (AMS) framework [19,32] and mapped onto Self-Determination Theory (SDT) regulations [9,13,33]. Intrinsic Motivation (AI-enhanced) was assessed with 2 items (e.g., “The availability of AI makes me even more curious to learn things myself”), reflecting interest/enjoyment and challenge despite AI’s presence [9,13].
- **Identified Regulation** (Value Alignment) was assessed with 2 items—“Even with AI, I still study because I value understanding things for myself” and “I want to use AI to support, not replace, my own learning process”—capturing personal endorsement of learning goals and deliberate, self-directed AI use [33,11].
- **External/Controlled Regulation** was assessed with 2 items—“I use AI tools mainly to get tasks done faster so I can meet deadlines” and “I feel pressure to use AI to stay competitive with other students”—reflecting reasons driven by external demands or peer comparison [9,13].
- Finally, Amotivation was captured by a single item, “Sometimes I wonder why I’m still doing the work myself when AI can do it for me,” indexing AI-related loss of purpose or volition [13,19]. Although the construct was represented by a single item, it was retained to ensure content coverage, consistent with prior evidence indicating that single-item indicators may be acceptable for concrete and unidimensional constructs within validation research contexts [34,35].Higher scores on intrinsic/identified items indicate more self-determined motivation in the presence of AI, whereas higher scores on controlled or amotivation items indicate greater external control or attenuated volition due to AI.
- **Basic Psychological Needs Satisfaction/Frustration – AI Context.** Guided by Self-Determination Theory [9,13] and the distinction between need satisfaction and frustration [20,36], a standard Basic Psychological Needs battery was extended with AI-context items to capture how generative AI may shape students’ sense of autonomy and competence during study. As part of a larger Basic Psychological Needs scale [20], four additional items targeting AI-specific influences on autonomy and competence were included. Students rated these on a 5-point scale (1 = Not at all true, 5 = Completely true). The items were:
- “I feel more in control of my learning when I use AI tools” (Autonomy satisfaction – AI context)
- “I feel pressure to use AI tools in ways that don’t reflect my personal values” (Autonomy frustration – AI context)
- “Using AI helps me feel more capable in my academic tasks” (Competence satisfaction – AI)
- “Relying on AI makes me doubt my own academic abilities” (Competence frustration – AI)

These items were designed to complement the standard need items (which assess general feelings of autonomy and competence in studies) by specifically probing AI-related experiences aligned with SDT’s theorization of volition, internalization, and perceived competence [9,13]. Although included in the AIM–N item pool, each of these was treated as a single-item indicator for its respective construct in confirmatory modeling (see Analytical Strategy), given that only one AI-context item was added per need domain. Consistent with recommended practice for single-indicator latent variables, each was specified as a reflective factor with the loading fixed to 1.00 and residual variance constrained as a function of an a priori reliability assumption (e.g., conservative ρ ≈.70), which yields identified and interpretable parameters without altering item content [21,37,38].

For completeness, the survey also administered other established scales (e.g., the Meaning in Life Questionnaire and its academic adaptation, and the remaining Basic Needs items from BPNSFS) to explore nomological validity. However, those results are beyond the scope of this paper, which focuses on the new AIM-N components.

#### AI usage frequency.

Participants reported “How often do you use AI tools for your academic work?” on a 5-point scale (Never, Rarely, Sometimes, Often, Very Often). This variable was used as a grouping factor in measurement invariance testing and as a continuous (ordinally treated) covariate within the MIMIC models. Additionally, participants indicated the purposes for which they use AI (multiple-choice) and how useful and ethical they perceive their AI use, but these were used mainly for sample description. In this study’s sample, 6% reported Never using AI, 24% Rarely, 35% Sometimes, 24% Often, and 12% Very Often, consistent with broad variation in AI engagement.

#### Field of study.

Categorical variable representing the student’s major field/discipline (e.g., Computer Science, Business, Humanities). For analysis purposes, the field of study variable was effect-coded in two ways: (1) as a set of dummy-coded indicators for use in MIMIC modeling, with Psychology designated as the reference category due to its large sample size, and (2) alternatively as a dichotomous contrast between STEM and non-STEM fields, which was applied in exploratory comparisons where differences between technical and non-technical domains were observed.

#### Gender.

Coded dichotomously for analysis (0 = female, 1 = male), excluding 18 respondents who chose “Prefer not to say” from gender-based subgroup analyses.

#### Study level.

Coded as 0 = undergraduate, 1 = postgraduate (combining Master’s/PhD) for certain analyses, or treated as three groups (Bachelor’s, Master’s, Doctoral) in multi-group CFA. Because relatively few participants were doctoral students (n = 32), invariance testing by study level compared undergraduates vs. postgraduates primarily, with PhD participants included with postgraduates for robustness when needed.

### Analytical strategy

All analyses were conducted in R and Python using appropriate packages for structural equation modeling and statistics. Because AIM–N is a newly developed instrument, an exploratory factor analysis (EFA) was conducted prior to confirmatory modeling to identify its latent structure, following established scale-development guidelines.

EFA was conducted on a different sample (40% of the initial one) to identify the latent structure of a scale measuring perceptions of artificial intelligence (AI) in academic contexts.

EFA was performed using principal axis factoring with Promax rotation, as factors were theoretically expected to correlate. Sampling adequacy was excellent (KMO =.870), and Bartlett’s test of sphericity was significant, χ²(45) = 816.86, p <.001, indicating that the correlation matrix was factorable.

The number of factors was evaluated using multiple criteria, including Kaiser’s eigenvalue-greater-than-one rule, inspection of the scree plot, and parallel analysis. Converging evidence supported a four-factor solution, corresponding to (a) Task-Level Redundancy, (b) Motivational/Identity-Level Redundancy, (c) AI-Related Motivational Orientations, and (d) AI-Context Need-Related Items.

All retained items showed salient loadings (≥.40) on their primary factor, with limited cross-loadings.

Confirmatory factor analysis (CFA) was then conducted on the AIM–N items. The hypothesized measurement model treated each theorized subscale as a latent factor. Based on the design, five latent factors were specified: Task-Redundancy (5 indicators), Motivational-Redundancy (5 indicators), Intrinsic Motivation (2 indicators), Identified Motivation (2 indicators), and Controlled Motivation (2 indicators). The single amotivation item and the four AI-context need items were not used as separate factors; the amotivation item was included in the CFA to see its loading (freely associated with the Controlled factor), but was dropped from the final model due to its low communality (see Results). Each latent factor was permitted to correlate with the others, as theoretical expectations suggested, for example, that redundancy beliefs would correlate positively with controlled motivation and negatively with intrinsic motivation.. Given the ordinal nature of the item responses (using 5-point and 7-point Likert scales), estimation was conducted using a robust weighted least squares estimator (WLSMV), incorporating a polychoric correlation matrix and robust (Huber–White) standard errors. Model fit was evaluated with standard indices: the Comparative Fit Index (CFI) and Tucker-Lewis Index (TLI) ≥ 0.95 indicating good fit, Root Mean Square Error of Approximation (RMSEA) ≤ 0.06, and Standardized Root Mean Square Residual (SRMR) ≤ 0.08, following conventional cutoffs. Modification indices were examined for localized misfit, but model modifications were only made if theoretically justified. Cronbach’s α and McDonald’s ω were computed for each multi-item subscale to assess internal consistency. Cronbach’s α was calculated using the standard variance formula, and McDonald’s ω was computed from the CFA factor loadings (reflecting the proportion of total variance explained by the factor) – these often converge with α for essentially unidimensional scales.

Next, measurement invariance of the AIM-N across the four grouping variables were tested: gender, study level, field, and AI usage frequency. For each grouping variable, a series of multi-group CFA models was estimated: (1) Configural invariance – the same factor structure freely estimated in all groups; (2) Metric invariance – factor loadings constrained equal across groups; and (3) Scalar invariance – loadings and item intercepts (thresholds for ordinal items) constrained equal across groups. Residual variances were left unconstrained, consistent with prior guidance that recognizes known differences in variability between groups. The primary focus remained on the invariance of measurement parameters rather than total variance equality. Measurement invariance was evaluated based on changes in model fit indices, with a change in Comparative Fit Index (ΔCFI) of ≤ –0.010 and a change in Root Mean Square Error of Approximation (ΔRMSEA) of ≤ + 0.015 used as thresholds to indicate non-significant decrement in model fit when progressing from less to more constrained models, in line with established recommendations.

In cases where full scalar invariance was not supported, modification indices were examined to identify items contributing substantially to intercept non-invariance. When justified, a single intercept was allowed to vary freely to achieve partial scalar invariance, consistent with best practices in multi-group confirmatory factor analysis. Such modifications were applied sparingly and are noted in the results section.

Finally, a Multiple Indicators, Multiple Causes (MIMIC) model was specified to examine both structural associations and differential item functioning (DIF) within a single integrated analysis. In this model, the latent factors previously identified through confirmatory factor analysis (CFA) were regressed on three observed covariates: AI usage frequency, gender, and field of study. These covariates were dummy-coded or effect-coded according to standard procedures. Although study level was also considered, it was excluded from the final model due to its high correlation with age and field of study; including it did not appreciably alter the estimates of other paths, and was omitted for the sake of parsimony.

To detect DIF, direct effects from the covariates to individual item indicators were incorporated into the model. Initially, a no-DIF MIMIC model was estimated, and modification indices were examined to identify potential DIF effects. Statistically significant direct paths (p <.05) that also demonstrated theoretical and substantive plausibility were retained in the final model. For example, a direct effect from field of study to the item “I feel pressure to use AI to stay competitive” was retained, as it was anticipated that students from highly competitive disciplines (e.g., Business or Computer Science) might exhibit elevated item responses beyond their general level of controlled motivation.

**Fig 1:**
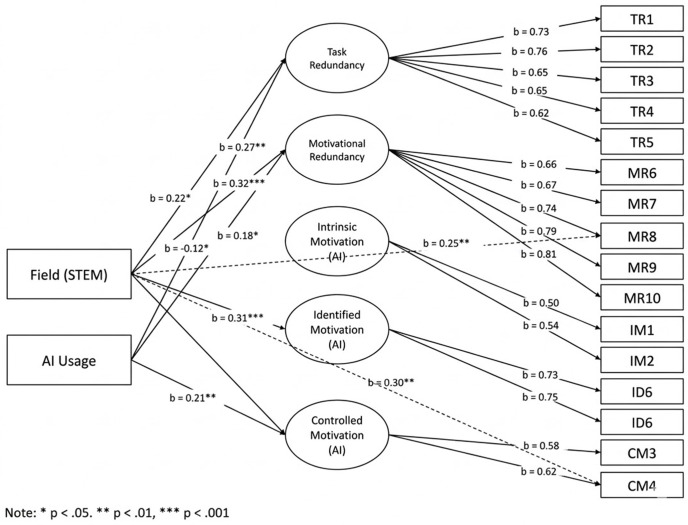
Conceptual diagram of the MIMIC model for AIM–N. Note. Latent factors (circles) indicated by their items (subset shown for clarity); observed covariates (squares: gender, study level, field, AI usage) predict the latent factors. The dashed arrow illustrates a potential DIF path (covariate → item) representing an intercept shift beyond the latent trait. Diagram is schematic and not to scale; no coefficients are shown here. Abbreviations: AIM–N, MIMIC, DIF.

**Fig 2:**
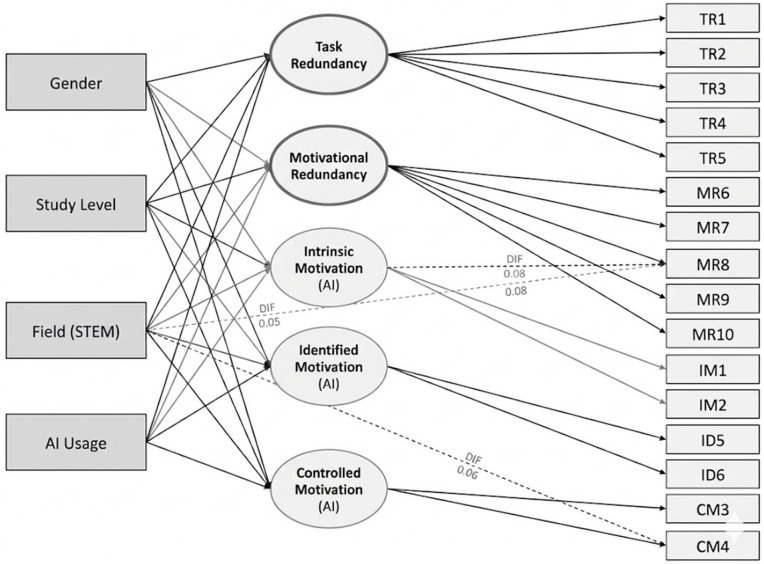
MIMIC model results for AIM–N (standardized coefficients). Note. Standardized path coefficients (β) are displayed only for significant predictors (*p* <.05); non-significant paths omitted for clarity. Dashed arrow denotes a retained DIF effect (e.g., Field → “stay competitive”/ “degree losing value”). Model fit for the results diagram: *χ*²(270) = 603.5, CFI =.958, RMSEA =.050; N = 904. Abbreviations: DIF = Differential Item Functioning. See Table 4 for numerical B, SE, β and exact *p*-values.

Fig 1 illustrates the conceptual structure of the MIMIC model, and Fig 2 presents the final model including all statistically significant paths. Both unstandardized and standardized coefficients are reported for structural relations. Model fit was evaluated using the same indices applied to the CFA. All statistical tests were conducted as two-tailed tests with an alpha level set at.05.

## Results

### Descriptive statistics and reliabilities

Descriptive statistics for the AIM-N subscales are presented in Table 1. Overall, students’ agreement with AI-Redundancy Beliefs was low-to-moderate. On the 5-point scale, the Task-Level Redundancy subscale (e.g., “learning certain skills is unnecessary now that AI can handle them”) had a mean of 1.92 (SD = 0.76), indicating most students disagreed that day-to-day learning tasks are pointless due to AI. The Motivational/Identity Redundancy subscale (e.g., “I worry my academic skills will soon be irrelevant because of AI”) had a slightly higher mean of 2.19 (SD = 0.80), suggesting some students have concerns about AI devaluing their overall degree or skills. Both redundancy subscales were right-skewed (skew = +0.56 and +0.27, respectively), with most responses clustering at the low end (“Strongly Disagree” or “Disagree”). Internal consistencies were high: Cronbach’s α = 0.87 and 0.83 for the task-level and motivational-level redundancy subscales, respectively, and McDonald’s ω were similarly 0.88 and 0.84. If combined into a single 10-item scale, redundancy beliefs showed α = 0.90. The two-factor structure was retained for theoretical clarity, as the two constructs were substantially correlated (r = 0.71) but exhibited evidence of differential conceptual emphasis, as indicated by the confirmatory factor analysis results.

**Table 1:** Descriptive statistics and reliability for AIM-N subscales (N = 904).

Subscale (No. of items)	Scale Range	Mean (SD)	Skewness	Kurtosis	Cronbach’s α	McDonald’s ω^a^
**Task-Level Redundancy** (5 items)	1–5	1.92 (0.76)	+0.56	–0.41	0.87	0.88
**Motivational Redundancy** (5 items)	1–5	2.19 (0.80)	+0.27	–0.65	0.83	0.84
**Intrinsic Motivation (AI)** (2 items)	1–7	4.77 (1.41)	–0.39	–0.24	0.43	0.45
**Identified Motivation (AI)** (2 items)	1–7	5.88 (1.25)	–1.40	+1.87	0.70	0.72
**Controlled Motivation (AI)** (2 items)	1–7	3.13 (1.56)	+0.39	–0.49	0.52	0.53
*Amotivation (AI)* (1 item)	1–7	3.95 (1.88)	–0.12	–1.25	–	–
*AI-Autonomy Satisfaction* (1 item)	1–5	3.30 (1.06)	–0.29	–0.51	–	–
*AI-Autonomy Frustration* (1 item)	1–5	2.51 (1.09)	+0.40	–0.74	–	–
*AI-Competence Satisfaction* (1 item)	1–5	3.12 (1.08)	–0.13	–0.73	–	–
*AI-Competence Frustration* (1 item)	1–5	2.84 (1.07)	+0.12	–0.81	–	–

**Note.** AIM–N subscales: **Task Redundancy** and **Motivational/Identity Redundancy** (5-point scale: Strongly Disagree–Strongly Agree); **Intrinsic Motivation (AI)**, **Identified Motivation (AI)**, **Controlled Motivation (AI)** (7-point scale); single items: **Amotivation (AI-induced)** and AI-context **Autonomy/Competence** need items (5-point). Reported metrics: M, SD, skew, kurtosis, Cronbach’s **α**, McDonald’s **ω** (multi-item subscales). For two-item subscales, also report Spearman–Brown reliability in the Supplement; inferential analyses rely on latent models. Higher scores indicate stronger endorsement. Abbreviations: **AIM–N** = AI-Motivation and Needs. *(See narrative description and reliability values in Results.)*

For the AI-Related Motivational Orientations (7-point scales), students reported mixed experiences. Identified motivation had the highest mean (5.88, SD = 1.25), implying strong endorsement of statements like “I want to use AI to support, not replace, my learning”. This subscale was negatively skewed (–1.40), with many students choosing “Corresponds a lot” or “Corresponds exactly” – a ceiling effect reflecting that most value learning for its own sake even with AI present. Intrinsic motivation under AI had a moderate mean of 4.77 (SD = 1.41), around the midpoint (“Moderately true for me”). Students on average agreed that AI makes them curious to learn and enjoy challenging themselves, though not uniformly strongly. Controlled motivation was the lowest, with mean 3.13 (SD = 1.56), indicating most students disagreed that they use AI due to external pressure or solely to meet requirements. This subscale was mildly right-skewed (+0.39). The internal consistency of the 2-item identified subscale was acceptable (α = 0.70; r_between items = 0.54). The intrinsic and controlled subscales, each based on 2 items, had lower reliabilities (α = 0.43 for intrinsic with item correlation r = 0.27; α = 0.52 for controlled, r = 0.35). These lower α values are not unexpected given the broad conceptual scope of the paired items (e.g., the two intrinsic items tapped curiosity vs. enjoyment of challenge, which are related but not identical facets). McDonald’s ω for these subscales (treating them as congeneric two-item factors in CFA) were 0.45 (intrinsic), 0.72 (identified), and 0.53 (controlled). Although the internal consistency estimates for the intrinsic and controlled motivation subscales were modest, these constructs were retained due to their theoretical significance within the self-determination framework. This limitation is addressed further in the discussion. Descriptive statistics for the single-item measure of AI-amotivation indicated a sample mean of 3.95 (SD = 1.88). Notably, responses to this item—“Why do it myself if AI can do it?”—exhibited substantial variability: approximately 25% of students agreed (rating 5 or above), while 50% firmly disagreed (rating 3 or below), with the remaining responses near the neutral midpoint. These distributions suggest that although a minority of students reported high levels of demotivation due to AI, the majority continued to perceive value in completing academic tasks independently.

Regarding the AI-context need items (treated individually), mean agreement was modest. For example, “Using AI helps me feel more capable” averaged 3.12/5 (SD = 1.08), indicating a slight positive effect on competence for some. “Relying on AI makes me doubt my abilities” averaged 2.84 (SD = 1.07), suggesting that many students did not feel undue self-doubt from AI, though a subset did. The autonomy-related items showed a similar pattern: “I feel more in control of my learning when I use AI” (mean 3.30, SD 1.06) and “I feel pressure to use AI in ways against my values” (mean 2.51, SD 1.09). These items did not form multi-item scales, so α was not reported; instead, they were examined via factor loading and DIF analysis in the structural model. In brief, redundancy beliefs correlated negatively with intrinsic and identified motivation (around r = –.30) and positively with controlled motivation (r ≈ +.35), aligning with SDT expectations that amotivation/low value accompanies more external regulation. Identified and intrinsic motivation correlated positively (r ≈ +.45), while controlled motivation was largely uncorrelated with intrinsic (r ≈ –.05) but had a small negative correlation with identified (r ≈ –.15), suggesting that valuing learning is somewhat incompatible with feeling externally pressured, in this context.

The exploratory factor analysis indicated a four-factor solution (Table 2), which was theoretically interpretable and broadly consistent with the measurement model subsequently tested via confirmatory factor analysis (CFA) in the remaining subsample. The first factor, Redundancy Beliefs – Task-Level, reflects students’ perceptions that specific academic tasks and learning activities have become less necessary due to AI’s capabilities. Items with the strongest loadings on this factor refer to questioning the need to learn certain content, viewing coursework as a formality, and finding it difficult to stay motivated when AI could easily complete the tasks. The second factor, Redundancy Beliefs – Motivational/Identity, captures broader concerns about the value of academic credentials and the future relevance of one’s skills in an AI-shaped environment; items loading strongly here include worry about degree devaluation and anticipated skill obsolescence. The third factor, AI-Related Motivational Orientations, represents a motivational conflict regarding the value of personal effort in learning when AI is perceived as capable of producing comparable or superior outcomes. Finally, the fourth factor, AI-Context Basic Needs Items, was comparatively less distinct, but appears to reflect AI-related perceptions linked to basic psychological needs (e.g., autonomy and competence), particularly where AI changes how learners experience agency and capability in academic work. Overall, the EFA supports a multidimensional representation of AI-related motivational and meaning-related perceptions in higher education, providing an empirical foundation for confirmatory validation.

**Table 2:** Exploratory factor analysis (EFA) for the identification of the latent structure of AIM-N scale.

Item	Factor 1: Redundancy Beliefs – Task-Level	Factor 2: Redundancy Beliefs – Motivational/Identity	Factor 3: AI-Related Motivational Orientations	Factor 4: AI-Context Basic Needs Items
1. I sometimes feel that my academic tasks are pointless because AI can complete them better or faster.	0.233	0.210	0.813	0.098
2. When working on assignments, I often wonder whether there’s any value in doing it myself if AI could do it for me.	0.366	0.148	0.706	0.010
3. The availability of AI tools makes me question whether I truly need to learn some of the things I’m being taught.	0.782	0.154	0.197	0.025
4. I feel like learning certain academic skills is unnecessary now that AI can handle them.	0.740	0.234	0.181	−0.074
5. Using AI makes me feel that much of my coursework is just an exercise in formality.	0.734	0.187	0.318	0.170
6. I find it hard to stay motivated in courses where AI could easily complete the main tasks.	0.653	0.297	0.326	0.215
7. AI tools have made some of my learning experiences feel redundant or outdated.	0.581	0.329	0.249	0.409
8. Sometimes I feel that my degree is losing its value because AI can do many of the things I’m being trained to do.	0.227	0.748	0.154	0.132
9. I would learn more enthusiastically if I believed AI tools weren’t already capable of doing what I’m learning.	0.216	0.693	0.174	0.259
10. I worry that the academic skills I’m developing will soon be irrelevant because of advancements in AI.	0.182	0.808	0.130	−0.155

**Note.** Extraction method: Principal Axis Factoring. Rotation method: Promax (oblique) withKaisernormalization. Rotation converged in 4 iterations.

### Factor structure interpretation

Factor 1: Redundancy Beliefs – Task-Level. This factor captures perceptions that specific learning tasks have become unnecessary or redundant due to AI’s functional capacity. The highest loadings included Item 3 (.782), Item 4 (.740), Item 5 (.734), Item 6 (.653), and Item 7 (.581). Conceptually, this factor reflects cognitive devaluation of academic tasks, suggesting that learners increasingly perceive traditional educational activities as less meaningful or less required in AI-rich learning contexts.

Factor 2: Redundancy Beliefs – Motivational/Identity. This factor reflects broader concerns regarding academic identity, longer-term motivation, and the perceived value of education under AI advancement. The strongest loadings included Item 10 (.808), Item 8 (.748), and Item 9 (.693). This dimension appears to capture existential and future-oriented concerns, where students worry that their educational investment (and degree value) may be undermined as AI advances.

Factor 3: AI-Related Motivational Orientations. This factor centers on learners’ internal conflict regarding effort and self-agency when AI is perceived as able to perform academic work efficiently or effectively. The strongest loadings included Item 1 (.813) and Item 2 (.706). This factor reflects AI-driven motivational disengagement, characterized by questioning the value of personal effort and decreased perceived justification for completing academic tasks independently.

Factor 4: AI-Context Basic Needs Items. This factor included items with more moderate loadings associated with psychological need-relevant experiences in AI-mediated learning. This factor was less sharply defined than the first three, with evidence of overlap (e.g., Item 7 showed cross-loading with Factor 1 at.409). Substantively, this factor may reflect AI-related experiences connected to autonomy and competence perceptions, especially in contexts where AI alters students’ felt control, capability, or the perceived purpose of learning activities.

#### Confirmatory Factor Analysis (CFA).

Aconfirmatory factor analysis (CFA) model was tested, as described in the Method section. This model yielded an adequate fit to the data: χ²(125) = 312.4, p <.001; CFI = 0.962, TLI = 0.949; RMSEA = 0.051 (90% CI [0.044, 0.058]); SRMR = 0.042. All items loaded strongly on their intended factors (standardized loadings ranged from 0.62 to 0.81 for the Redundancy items, 0.50–0.54 for Intrinsic, 0.73–0.75 for Identified, and 0.58–0.62 for Controlled; see Table 4). The only exception was the single amotivation item, which was provisionally associated with the Controlled Motivation factor – its loading was low (0.32) and non-significant, indicating it did not share sufficient variance with the two controlled-regulation items. This pattern suggests that the item reflecting “wondering why one should do the work” aligns more closely with a general factor of controlled motivation. Consequently, the amotivation item was excluded from the confirmatory factor analysis and treated instead as an auxiliary single-item measure for descriptive and exploratory purposes. After removing that item, model fit improved slightly (CFI = 0.968, TLI = 0.958, RMSEA = 0.047). The factor correlation matrix is shown in Table 2. As expected, the two Redundancy factors were highly correlated (r = 0.81), justifying an alternative single-factor model for redundancy. The model tested collapsed them into one factor; this one-factor redundancy model had slightly worse fit (Δχ²(1) = 45.8, p <.001; CFI dropped to 0.953), and modification indices suggested the two-factor solution better accounts for a minor split in content (items about specific tasks versus broader academic trajectory). Given the theoretical meaningfulness of that split, the two-factor structure was retained for redundancy beliefs in subsequent analyses. The three motivation factors showed an interesting pattern: Identified and Intrinsic motivations were moderately positively correlated (r = 0.48), whereas Controlled motivation was essentially uncorrelated with Intrinsic (r = –0.04, n.s.) and had a small negative correlation with Identified (r = –0.22). This indicates that students who strongly value learning for themselves (high identified) or enjoy learning (intrinsic) did not tend to feel externally pressured to use AI. Those relationships align with SDT’s notion that self-determined vs. controlled motivations are distinct dimensions, even though they are not strongly inverse here. The Redundancy beliefs factors were negatively correlated with both Intrinsic and Identified motivation (r ≈ –0.35 to –0.40, p <.001), supporting the idea that seeing less meaning in learning due to AI goes hand-in-hand with lower self-driven motivation. Redundancy beliefs were positively correlated with Controlled motivation (r ≈ +0.38), implying that students who feel AI makes learning pointless are also more likely to feel pressure or external regulation in their studies. Overall, the CFA supports the multidimensional structure of AIM-N and provides evidence of construct validity through these inter-factor correlations.

Factor loadings and modification indices related to the four AI-context need satisfaction/frustration items, were also examined. These items were not part of the main CFA model since each stood alone. However, an exploratory factor analysis on the need items (with standard need items included) indicated that the AI-based autonomy and competence items loaded strongly with their respective need frustration/satisfaction factors (e.g., “I feel more in control with AI” loaded with other Autonomy Satisfaction items at 0.59; “Relying on AI makes me doubt abilities” cross-loaded with Competence Frustration at 0.52). This suggests these items tap the intended need dimensions but also contain unique variance. For the sake of parsimony, these standalone items were not incorporated as additional indicators in the final confirmatory factor analysis (CFA) model of the AIM–N. This decision was made to avoid conflating context-specific effects with the measurement of general psychological need factors. Instead, potential item-level effects were assessed through MIMIC-based differential item functioning (DIF) analysis. In summary, the CFA results supported the proposed five-factor structure of the AIM–N (excluding the standalone items), demonstrating acceptable model fit and theoretically meaningful factor correlations.

### Measurement invariance

Measurement invariance of the AIM–N factor structure was evaluated across multiple grouping variables. As shown in Table 4, fit indices are presented for configural, metric, and scalar invariance models tested for each grouping. These tests were conducted using the final five-factor model, which included two redundancy factors and three motivation factors.

#### Gender invariance.

The configural model (freely estimating the same 5-factor model in males vs. females) showed good fit (CFI = 0.963, RMSEA = 0.049). Constraining loadings equal (Metric) had ΔCFI = –0.002 and ΔRMSEA = +0.001, indicating metric invariance held. Constraining intercepts (Scalar) resulted in ΔCFI = –0.004 and ΔRMSEA = +0.002, still within recommended criteria. Thus, full scalar invariance by gender was supported. No evidence of gender-based differential item functioning (DIF) was observed, indicating that male and female respondents interpreted and responded to the AIM–N items in an equivalent manner. Latent mean comparisons similarly revealed no significant gender differences on any of the latent factors, consistent with the structural path estimates presented below.

#### Study level invariance.

Following the initial model evaluation, measurement invariance was subsequently tested across Bachelor’s and Postgraduate student groups to examine the stability of the factor structure across academic levels. (Doctoral students were too few for a standalone group; they were included with postgraduates for this test, after verifying configural similarity.) Configural fit was acceptable (CFI = 0.959). Metric invariance held (ΔCFI = –0.003). For scalar invariance,a slight drop in fit (ΔCFI = –0.011), was observed. The largest modification index suggested that item 8 (“Sometimes I feel my degree is losing value because AI can do many of the things I’m being trained to do”) had different intercepts between undergrads and grads – undergraduates tended to agree somewhat more at the same level of the latent redundancy factor. The intercept for this item was allowed to vary between groups (partial scalar invariance), resulting in an improved model fit (CFI increased to 0.956; ΔCFI = –0.003 relative to the metric model). With this adjustment, scalar invariance was established for all other items in the model. This finding indicates that, apart from the item in question—which was subsequently addressed as a case of differential item functioning (DIF) within the MIMIC model—the AIM–N instrument functions equivalently across undergraduate and graduate student groups.

#### Field of study invariance.

Testing invariance across all ten specified fields was challenging due to small N in some categories (e.g., n = 6 in Law). Fields of study were grouped into five broad categories for the purposes of invariance testing.: Tech (Computer Science, Engineering; n = 106), Science (Natural & Health Sciences; n = 194), Social Science (Psychology, Social Sciences; n = 228), Humanities/Arts (Humanities, Law; n = 45), and Business/Economics (n = 110). This grouping balances sample sizes and conceptual similarity. The 5-group configural model fit was borderline (CFI = 0.941, RMSEA = 0.054), likely due to the small Humanities group. Still, loadings were all significant in each group and no major model misspecification appeared. Imposing metric invariance across fields yielded ΔCFI = –0.006, indicating factor loadings can be considered equal. Scalar invariance across five groups was more difficult: the initial scalar model had ΔCFI = –0.020, failing the criterion. Modification indices highlighted two items with intercept differences: one Redundancy item (“my degree is losing value…” again) and one Controlled motivation item (“I feel pressure to use AI to stay competitive”). For the degree-value item, students in Tech fields showed higher agreement intercept, whereas for the competitive-pressure item, Business students showed higher intercepts, after controlling for latent factors. Two item intercepts were freely estimated to achieve partial scalar invariance. This adjustment reduced ΔCFI to –0.008, indicating that partial invariance was acceptable according to established criteria. With the exception of these specific items—addressed further through MIMIC modeling—the AIM–N factor structure was found to be invariant across academic fields.

#### AI usage frequency invariance.

Invariance was subsequently examined across the five self-reported AI-use frequency groups (Never, Rarely, Sometimes, Often, Very Often). The configural model demonstrated an acceptable fit (CFI = 0.952, RMSEA = 0.052). Factor loadings appeared similar across groups; metric invariance was supported (ΔCFI = –0.005). Scalar invariance, however, was not fully supported across five groups (ΔCFI = –0.018). The “Not at all true/Completely true” 5-point items in particular showed some threshold shifts for the Never users group, which had a restricted range (many “Never” users simply answered neutral for lack of experience). Two item intercepts were allowed to vary for participants in the “Never” group. One of these concerned an autonomy-related AI item (“I feel more in control when using AI”), which was frequently marked low by participants who reported never using AI—likely reflecting the absence of relevant experience rather than underlying motivational disposition. The second concerned a redundancy-related item (“I find it hard to stay motivated in courses where AI could easily complete the tasks”), which also tended to receive lower scores from non-users of AI, potentially because those participants had not directly encountered the efficiency-related implications of AI use in coursework. These intercept adjustments were made to account for group-specific response patterns not attributable solely to latent factor variation. With those freed, partial scalar invariance was achieved (ΔCFI = –0.009). This pattern was interpreted as evidence that the scale functioned similarly among students who reported at least occasional AI use (i.e., rarely, sometimes, or often). However, caution is warranted when interpreting responses from individuals who reported never using AI tools. For these respondents, certain item statements may have been perceived as hypothetical or abstract, potentially influencing their response patterns. In the subsequent MIMIC analyses, AI usage frequency was included as a continuous covariate to account for these individual differences in experience with AI.

In summary, the AIM-N demonstrated strong measurement invariance properties. Table 3 provides the key fit indices and ΔCFI for each step. These results suggest that group comparisons (e.g., latent mean differences) are meaningful – any observed differences reflect true differences in the underlying constructs rather than measurement artifact. This established invariance permitted the comparison of latent factor means across groups, with results reported in the Discussion section for interpretative purposes.

**Table 3:** Correlations among AIM-N Latent Factors (CFA).

Latent Factor	1. Task Redundancy	2. Motivational Redundancy	3. Intrinsic Mot. (AI)	4. Identified Mot. (AI)	5. Controlled Mot. (AI)
**1. Task Redundancy**	–				
**2. Motivational Redundancy**	0.81***	–			
**3. Intrinsic Motivation**	–0.35***	–0.39***	–		
**4. Identified Motivation**	–0.36***	–0.40***	0.48***	–	
**5. Controlled Motivation**	0.38***	0.34***	–0.04	–0.22**	–

**Note.** Correlations are standardized estimates from CFA (N = 904). *p <.001, p <.01. All are two-tailed. The single-item Amotivation measure is excluded here (not a latent factor). Entries are Pearson **r** among observed subscale scores (two-tailed); significance: *p* <.05, **p** <.01, ***p*** <.001. Latent factor correlations (from CFA) are standardized and presented in a separate panel/row. AIM–N subscales defined as in Table 1.

**χ²(df)**, **CFI**, **RMSEA** (90% CI), and **ΔCFI** for **Configural**, **Metric (Λ equal)**, and **Scalar (Λ,τ equal)** models across groups (**Gender**, **Study Level**, **Field of Study**, **AI-Use Frequency**). Invariance judged by ΔCFI ≥ –.010 and ΔRMSEA ≤ +.015 when moving to more constrained models. Where **partial scalar** was used, freed intercepts are indicated by footnotes **a–c** (see table): e.g., “degree losing value” and “stay competitive” items freed for specific groups/fields; Never-use group with freed thresholds on two items. Abbreviations: **CFI** = Comparative Fit Index; **RMSEA** = Root Mean Square Error of Approximation; **ΔCFI** = change in CFI. Criteria and freed-parameter details correspond to those reported in the Results text.

In addition to absolute fit indices, the chi-square to degrees of freedom ratio (χ²/df) is reported as a descriptive indicator of relative model fit. Across all grouping variables and invariance levels, χ²/df values ranged from approximately 1.26 to 1.76, which is generally considered indicative of acceptable to good model fit in structural equation modeling. Given the sensitivity of the chi-square statistic to large sample sizes, χ²/df is reported here for descriptive purposes only, while changes in comparative fit indices (ΔCFI) and RMSEA were used as the primary criteria for evaluating measurement invariance. Consistent χ²/df values across configural, metric, and (partial) scalar models further support the overall stability of the measurement structure across groups.

### MIMIC model: Structural paths and DIF

The grouping variables were incorporated into a single Multiple Indicators, Multiple Causes (MIMIC) model. This structural equation model included the five latent factors (as measured by their indicator items, using the CFA loadings from the invariant model) and regressed each factor on AI Usage Frequency, Gender, and Field of Study. Field of Study was effect-coded as a binary contrast (STEM = 1 vs. non-STEM = 0) to facilitate interpretation in the main model, given that the detected DIF primarily reflected distinctions between technical and non-technical academic disciplines. (In a supplementary MIMIC model with multiple field dummy variables, the strongest effects were indeed for Computer Science/Engineering vs. others.) Study Level was also included as a covariate initially; it showed no unique effects after accounting for age and field (e.g., postgraduates had slightly lower redundancy beliefs than undergrads, but this overlapped with field differences), so it was dropped to avoid multicollinearity. Gender was coded 0 = female, 1 = male. AI usage was treated as a continuous ordinal (1 = Never to 5 = Very Often). The MIMIC model fit well: χ²(270) = 603.5, CFI = 0.958, RMSEA = 0.050.

Fig 2 (results diagram) illustrates the significant paths. Table 5 presents the numerical estimates of all structural paths and any retained DIF direct effects. Standardized coefficients (β) are reported to facilitate interpretation, with corresponding unstandardized estimates (B) and standard errors (SE) presented in Table 5.

**Table 5:** MIMIC model regression paths and DIF Effects.

Predictor (Covariate)	Outcome (Dependent)	B (SE)	β (Standardized)	p-value
**AI Usage Frequency**	→ Task Redundancy factor	+0.237 (0.058)	+0.32 ***	<.001
	→ Motivational Redundancy	+0.201 (0.064)	+0.31 ***	0.001
	→ Intrinsic Motivation (AI)	+0.083 (0.072)	+0.06	0.246
	→ Identified Motivation (AI)	+0.094 (0.066)	+0.08	0.159
	→ Controlled Motivation (AI)	+0.208 (0.073)	+0.21 **	0.005
**Gender (Male = 1 vs F = 0)**	→ Task Redundancy	+0.055 (0.084)	+0.04	0.517
	→ Motivational Redundancy	+0.061 (0.089)	+0.05	0.493
	→ Intrinsic Motivation (AI)	–0.046 (0.103)	–0.02	0.655
	→ Identified Motivation (AI)	+0.027 (0.082)	+0.01	0.742
	→ Controlled Motivation (AI)	+0.118 (0.095)	+0.06	0.212
**Field (STEM = 1 vs non = 0)**	→ Task Redundancy	+0.384 (0.134)	+0.27 **	0.004
	→ Motivational Redundancy	+0.298 (0.139)	+0.22 *	0.029
	→ Intrinsic Motivation (AI)	–0.147 (0.140)	–0.09	0.298
	→ Identified Motivation (AI)	–0.182 (0.092)	–0.12 *	0.048
	→ Controlled Motivation (AI)	+0.295 (0.117)	+0.18 *	0.012
**Study Level (PG vs UG)**^a^	→ Task Redundancy	–0.214 (0.098)	–0.15 *	0.029
	→ (other factors n.s.)			
*DIF Direct Effects:*				
Field (STEM) → *“Stay competitive” item*	+0.453 (0.141)	+0.30 **	0.001	
Field (STEM) → *“Degree losing value” item*	+0.381 (0.123)	+0.25 **	0.002	
(no Gender or Usage DIF paths were significant)				

**Note.** Unstandardized coefficients **B** (SE) and standardized **β** from the Multiple Indicators Multiple Causes (**MIMIC**) model; **N** = 904. Covariates: **AI usage** (1 = Never to 5 = Very Often), **Gender** (0 = female, 1 = male), **Field** (effect-coded: **STEM** = 1 vs non-STEM = 0; in a supplementary model, finer dummies yielded the same pattern). Study Level was dropped from the final model due to overlap with field (see text). **p** values are two-tailed; significance: *p* <.05, **p** <.01, ***p*** <.001. **DIF** rows display direct effects from covariates to specific items retained on theoretical and statistical grounds (e.g., Field → “stay competitive”; Field → “degree losing value”). Non-significant paths are either shown as “ns” or omitted for clarity (as indicated in the table stub). Model fit for this MIMIC specification: χ²(270) = 603.5, **CFI** =.958, **RMSEA** =.050.

#### Effects of AI usage.

As anticipated, AI usage frequency was a significant predictor of certain motivational beliefs. Students who use AI more often tended to endorse higher AI-Redundancy Beliefs (β = +0.32, p <.001): in standardized terms, moving from one usage category to the next (e.g., Rarely to Sometimes) was associated with about one-third standard deviation increase in feeling that learning tasks are replaceable or meaningless. This aligns with the notion that extensive exposure to AI’s capabilities can lead students to devalue traditional learning activities. AI usage also had a significant positive effect on Controlled Motivation (β = +0.21, p =.005), suggesting heavy AI users more often cite external reasons (e.g., using AI to save time or due to peer pressure) for their study behavior. In contrast, usage frequency had no significant effect on Intrinsic Motivation (β = +0.06, n.s.) or Identified Motivation (β = +0.08, n.s.). In other words, students who frequently use AI are not significantly less (or more) intrinsically interested in learning, nor do they differ in valuing learning for its own sake, once other factors are controlled. This is an important finding: high AI use does not inherently erode all forms of motivation – its impact seems specific to amplifying feelings of task redundancy and some external regulation. (Notably, a bivariate correlation did show a mild negative correlation between AI use and intrinsic motivation, but in multivariate context that was accounted for by field differences – e.g., CS majors both use AI more and tend to have lower intrinsic motivation, see below.) AI usage also had no significant direct effect on the basic need satisfaction items in the MIMIC model; any such effect is presumably mediated via the latent factors (e.g., high AI users feel less autonomy not directly, but because they feel more externally controlled in their motivation).

#### Effects of gender.

Controlling for other variables, gender showed no statistically significant influence on any AIM-N factor. The path estimates for male (vs. female) were β = +0.04 for Redundancy, –0.02 for Intrinsic, + 0.01 for Identified, and +0.06 for Controlled (all p >.20). These very small differences align with this study’s earlier finding of measurement invariance and equal latent means by gender. It appears that male and female students in this sample are similarly motivated (or demotivated) in the context of AI. Although the gender-based coefficients were not statistically significant, the direction of the effects suggested that male participants scored slightly higher on controlled motivation and redundancy. This pattern is occasionally observed in technology-related studies, where males are sometimes described as more gadget-oriented or more likely to engage in efficiency-seeking behaviors. However, in the present context, these differences were negligible in magnitude and did not reach statistical significance. Consequently, gender-based differential item functioning (DIF) was not investigated further, as no evidence for such effects emerged from the confirmatory factor analysis. These findings support the interpretation that the AIM–N instrument functions in a gender-neutral manner within this sample.

#### Effects of field of study.

The field effects were analyzed with the STEM vs. non-STEM contrast (1 = STEM fields, defined here as Computer Science, Engineering, or closely related technical majors; 0 = others). This contrast was chosen based on the partial invariance results indicating technical fields showed some distinct response patterns. Indeed, field (STEM) emerged as a significant predictor for several factors. STEM students scored significantly higher on Redundancy Beliefs (β = +0.27, p =.004) than non-STEM students, after controlling for AI usage and other factors. This suggests that those in technology-related fields are more prone to feel that AI overtakes human learning (perhaps because they better understand AI capabilities, or their curricula involve tasks AI can handle). Additionally, STEM students showed lower Identified Motivation (β = –0.12, p =.048), indicating they are slightly less likely to personally value learning for its own sake in the presence of AI, compared to their peers in non-STEM fields. One possible interpretation is that some STEM students might view learning more instrumentally (e.g., focusing on efficiency and outcomes, which AI can provide) whereas humanities/social science students might emphasize personal meaning that AI cannot replace. Field had no significant effect on Intrinsic Motivation (β = –0.09, n.s.), though the coefficient was negative in sign. The strongest field effect was on Controlled Motivation – STEM students were considerably more likely to feel external pressure or use AI for deadline-oriented reasons (β = +0.18, p =.012). This could reflect a competitive culture in technical fields or higher normative pressure to adopt AI tools to keep up with peers (e.g., coding assistance tools in CS).

Further analyses were conducted to explore disciplinary differences in greater detail. Students enrolled in Computer Science and Engineering programs were found to report the highest levels of perceived redundancy and controlled motivation, whereas students from disciplines such as Humanities and Social Sciences exhibited the lowest scores on these constructs. Students in Psychology and Education typically scored in the mid-range across these dimensions. These observed trends were consistent with the binary STEM versus non-STEM contrast applied in the primary analyses. Accordingly, the STEM grouping was considered a reasonable representation of systematic variation in AI-related motivation across academic fields.

#### Effects of study level.

Although study level (undergraduate vs. postgraduate) was not included in the final model due to potential multicollinearity with age and field of study, an auxiliary model incorporating this variable indicated that postgraduate students reported slightly lower redundancy beliefs on average (β ≈ –0.15, p <.05). This finding suggests that graduate students were less likely to perceive AI as nullifying the value of learning. This difference mirrors the partial invariance finding for one item. It may be that more advanced students have a stronger commitment to their field or see the limitations of AI more clearly, reducing feelings that AI makes learning pointless. No other factor showed a significant difference by study level in that model. Since the inclusion of study level did not notably alter other parameter estimates, the more parsimonious model is presented in Table 5.

#### Differential Item Functioning (DIF).

The MIMIC framework allowed us to detect and estimate DIF as direct effects of covariates on item responses, beyond the latent factor. Through the examination of modification indices, two significant DIF paths were identified, both involving the contrast between STEM and non-STEM fields of study: (1) a direct effect on the Controlled Motivation item “I feel pressure to use AI to stay competitive with other students.” Even after accounting for the latent Controlled motivation factor, being in a STEM field added an extra B = +0.45 (SE = 0.14, p =.001) on that item’s 7-point response. Standardized, this was about β = +0.30 (see Fig 2 dashed path). This indicates a specific bias: STEM students agreed notably more with the “competitive pressure” statement than would be expected given their overall controlled motivation level. In contrast, non-STEM students with equivalent latent controlled motivation showed lower responses on that particular item. In practical terms, this DIF suggests that the competitive aspect of AI use is especially salient in STEM disciplines. The item intercept was allowed to vary to accommodate this effect, consistent with the partial invariance approach applied earlier. (2) The second DIF was a direct effect of STEM field on the Motivational Redundancy item “Sometimes I feel my degree is losing its value because AI can do many of the things I’m being trained to do.” STEM students scored higher on this item controlling for the redundancy factor (B = +0.38, SE = 0.12, p =.002; β ≈ +0.25). This suggests that, over and above general redundancy beliefs, STEM majors particularly worry about the value of their degree in an AI future. This may be attributable to the nature of training in those programs, which often emphasize technical skills perceived as more susceptible to automation by AI. In contrast, students in fields such as the social sciences may perceive that their human-centered competencies retain distinctive value. This DIF path was also retained in the final model (and corresponds to the freed intercept in invariance tests). No other item showed meaningful DIF by gender or usage after accounting for these. Notably, the Never AI users group differences were largely captured by the latent means (they scored lower on Redundancy factor itself), so no additional direct effect for “Never use” on items was needed beyond what was already freed in partial invariance (Two intercepts were freely estimated for the “Never-use” category in earlier CFA models. Including AI usage as a continuous covariate in the MIMIC model served to statistically adjust for this variation in a more parsimonious manner).

In sum, the MIMIC results highlight that the strongest predictors of feeling demotivated by AI were the behavioral exposure to AI and the academic context. High AI usage frequency is associated with more negative motivational perceptions (task redundancy, external regulation), and being in a technology-oriented field amplifies those perceptions. By contrast, demographic factors like gender or broad educational level were minimal influences. It was observed that certain survey items may carry distinct connotations depending on the academic field of respondents—an important nuance to be considered by practitioners utilizing the scale in diverse disciplinary contexts. (e.g., when interpreting the “competitive pressure” item, one should consider field context). With the DIF adjustments, however, the AIM-N can be used fairly across groups.

### Final AIM-N scale and factor loadings

Table 6 presents the finalized AIM-N item battery with their factor loadings from the CFA. The scale is designed to be scored either by subscale or in aggregate for certain constructs. Based on the results of the present analysis, it is recommended that the instrument be scored as follows: (a) compute separate means for the 5-item Task Redundancy and 5-item Motivational Redundancy subscales (or a total 10-item Redundancy score if unidimensionality is assumed for a given application, noting slightly lower precision), (b) compute means for the 2-item Intrinsic, 2-item Identified, and 2-item Controlled motivation subscales (with caution that Intrinsic and Controlled subscales have modest reliability and might be better used in combination or for group comparisons rather than high-stakes individual assessment), and (c) treat the single-item Amotivation and the four AI-context need items as standalone indicators or qualitative flags (e.g., high scores on the amotivation item signal a student who may be particularly disengaged due to AI). In this’study’s validated model, each multi-item subscale’s items had strong and significant loadings on their respective factors, indicating good convergent validity.

**Table 6:** AIM-N scale items, subscales, and standardized CFA Loadings.

Item (English text) (Likert scale)	Subscale/ Factor	Loading^b^
**Redundancy Beliefs – Task-Level** (5-point: *Strongly Disagree*–*Strongly Agree*)	*(Factor 1)*	
1. *“I sometimes feel that my academic tasks are pointless because AI can complete them better or faster.”*	Redundancy – Task	0.73***
2. *“When working on assignments, I often wonder whether there’s any value in doing it myself if AI could do it for me.”*	Redundancy – Task	0.76***
3. *“The availability of AI tools makes me question whether I truly need to learn some of the things I’m being taught.”*	Redundancy – Task	0.68***
4. *“I feel like learning certain academic skills is unnecessary now that AI can handle them.”*	Redundancy – Task	0.65***
5. *“Using AI makes me feel that much of my coursework is just an exercise in formality.”*	Redundancy – Task	0.62***
**Redundancy Beliefs – Motivational/Identity** (5-point scale, cont’d)	*(Factor 2)*	
6. *“I find it hard to stay motivated in courses where AI could easily complete the main tasks.”*	Redundancy – Motivational	0.66***
7. *“AI tools have made some of my learning experiences feel redundant or outdated.”*	Redundancy – Motivational	0.67***
8. *“Sometimes I feel that my degree is losing its value because AI can do many of the things I’m being trained to do.”*	Redundancy – Motivational	0.74***
9. *“I would learn more enthusiastically if I believed AI tools weren’t already capable of doing what I’m learning.”*	Redundancy – Motivational	0.79***
10. *“I worry that the academic skills I’m developing will soon be irrelevant because of advancements in AI.”*	Redundancy – Motivational	0.81***
**AI-Related Motivational Orientations** (7-point: *Does not correspond*–*Corresponds exactly*)	*(Factors 3–5)*	
1. *“The availability of AI makes me even more curious to learn things myself.”*	Intrinsic Motivation (AI)	0.50***
2. *“I enjoy challenging myself to solve academic problems without relying too much on AI.”*	Intrinsic Motivation (AI)	0.54***
3. *“I use AI tools mainly to get tasks done faster so I can meet deadlines.”*	Controlled Motivation (AI)	0.58***
4. *“I feel pressure to use AI to stay competitive with other students.”*	Controlled Motivation (AI)	0.62***
5. *“Even with AI, I still study because I value understanding things for myself.”*	Identified Motivation (AI)	0.73***
6. *“I want to use AI to support, not replace, my own learning process.”*	Identified Motivation (AI)	0.75***
7. *“Sometimes I wonder why I’m still doing the work myself if AI can do it for me.” (single item)*	Amotivation (AI-induced)	–^c^
**AI-Context Basic Needs Items** (5-point: *Not at all true*–*Completely true*)	*(Single items)*	
A. *“I feel more in control of my learning when I use AI tools.”*	Autonomy Satisfaction (AI)	–^c^
B. *“I feel pressure to use AI tools in ways that don’t reflect my personal values.”*	Autonomy Frustration (AI)	–^c^
C. *“Using AI helps me feel more capable in my academic tasks.”*	Competence Satisfaction (AI)	–^c^
D. *“Relying on AI makes me doubt my own academic abilities.”*	Competence Frustration (AI)	–^c^

**Note.** Items are grouped by subscale; entries are **standardized CFA loadings** (all *p* <.001). Single-item indicators (Amotivation and AI-context need items) were not modeled as multi-indicator factors in the final CFA (hence no loadings; see footnote **c** in table). Likert anchors: Redundancy & needs = 5-point; motivation = 7-point. Recommended scoring (summarized in text): means for each subscale; Redundancy can be kept as two 5-item subscales or combined (10-item) with a note about precision; two-item motivation subscales reported with caution for individual diagnostics. Abbreviations: **CFA** = Confirmatory Factor Analysis.

## Discussion

This study developed and validated the AIM–N scale, which fills a critical gap by measuring how the presence of AI technology in academic work relates to students’ motivation and basic psychological needs. Overall, this study’s findings demonstrate that the AIM–N is a psychometrically sound instrument with a clear factor structure, good reliability for most subscales, and measurement invariance across a range of student groups. The establishment of measurement invariance was considered essential for ensuring the validity of cross-group comparisons [23]. Evaluation of invariance was conducted using widely recommended criteria for multi-group comparisons [39], supplemented by best-practice guidelines addressing measurement bias and invariance in latent constructs [40]. In addition, the Multiple Indicators, Multiple Causes (MIMIC) framework was employed as a complementary approach for probing differential item functioning (DIF) while accounting for the influence of latent trait structures [41, 24]. This dual approach provided a robust analytic foundation for detecting both uniform and non-uniform sources of item-level bias across covariate-defined groups. Taken together, the results provide nuanced insights into student experiences in the AI era: some students indeed feel that AI undermines the meaning and value of their education (redundancy beliefs), and this outlook is associated with more externalized motivation; however, many students remain intrinsically or identified-motivated—for example, they enjoy learning for its own sake and want to use AI as a supportive tool rather than a replacement. These patterns align with contemporary perspectives on science/technology-enhanced learning and motivation.

### Interpreting identified-high vs. intrinsic-moderate

Identified regulation remained high, whereas intrinsic motivation under AI was more moderate and showed modest internal consistency across its two facets (curiosity vs. enjoyment of doing tasks without AI). Conceptually, AI may preserve **valued goals** (“understanding still matters”) while reducing **task-level enjoyment/challenge** by making routine work easier or more procedural. This differentiation aligns with SDT: personally endorsed reasons can persist even when the activity feels less inherently enjoyable. Instructionally, tasks may need **novelty, optimal challenge, and visible human value** to sustain intrinsic interest in AI-rich contexts.

### Factor structure of AIM–N and theoretical implications

The confirmatory factor analysis supported a multi-dimensional structure, indicating that the impact of AI on student motivation is not a single monolithic construct but can be differentiated into specific factors. Notably, the hypothesized bifactor structure of AI-related Redundancy Beliefs—differentiating Task Redundancy from Motivational Redundancy—was confirmed through confirmatory factor analysis: one concerning immediate learning tasks and another concerning the broader value of one’s education. This distinction mirrors classic concepts of amotivation at different levels within Self-Determination Theory (SDT): item content in the first factor reflects task-specific apathy (e.g., “Why do this assignment if AI can do it?”), whereas the second factor verges on existential academic doubt (e.g., “Is my degree worth anything if AI can do these jobs?”). Although the two factors were highly correlated (r ≈.80), the separation improved model fit and reliability. Practically, this suggests that while both reflect a loss of meaning, educators might address them differently—emphasizing the human value of the learning process to counter degree-level concerns, and re-designing assignments to counter task-level redundancy. The pattern resonates with SDT’s account that experiences of meaninglessness co-occur with amotivation [9,36] and with motivation work in education showing that value appraisals matter for sustained engagement [26]. As expected, the AIM–N Redundancy subscales correlated negatively with intrinsic and identified motivation, consistent with SDT’s view that amotivation stands in contrast to self-determined forms of motivation [9,19].

This study’s AI-Motivational Orientations subscales (Intrinsic, Identified, Controlled) also behaved largely as expected, with some informative nuances. The Identified regulation items (valuing learning itself despite AI) formed the strongest subscale, with high mean scores and loadings, suggesting many students still endorse the importance of learning even with AI available—echoing frameworks that tie sustained engagement to personally endorsed task value [26]. By contrast, Intrinsic motivation in the context of AI was moderate and its two indicators showed only modest inter-correlation: one item tapped curiosity spurred by AI (a counter-intuitive positive—“AI makes me more interested to learn myself”), while the other tapped enjoyment of non-AI challenge. The low correlation implies these are distinct facets of intrinsic motivation vis-à-vis AI—some students are intrigued by AI’s capabilities (curiosity) whereas others derive satisfaction from not using AI as a crutch. Future research might expand these items or use qualitative methods to clarify how AI shapes interest and enjoyment [36]. Finally, Controlled motivation (using AI due to pressure or to meet external demands) showed acceptable coherence and emphasises a potential downside: students may feel emerging social or institutional pressure to use AI to keep up. This aligns with SDT’s definition of controlled regulation [9,36] and with broader models in which subjective norms influence technology uptake [42,43]. Importantly, controlled motivation did not significantly correlate with intrinsic motivation (r ≈ −.04), indicating relative orthogonality in this context—students can feel pressure yet still enjoy learning, or vice versa. Controlled motivation did correlate positively (r ≈.38) with redundancy beliefs, hinting that external pressure to use AI may co-occur with perceiving tasks as pointless (because one pursues them to “get them done,” not for personal value).

The AI-context basic needs items indicated that AI’s effects on autonomy and competence are double-edged. Using AI made some students feel more capable (competence satisfaction), yet also led others to doubt their abilities (competence frustration). Likewise, some students felt more in control with AI—potentially because their learning could be steered more efficiently—while others felt pressured to use AI in ways they regarded as inappropriate, reflecting a frustration of autonomy. These contrasts align with the premise in Self-Determination Theory (SDT) that technologies can either support or thwart basic needs depending on affordances and context [9,13]. They also echo concerns about cognitive offloading and automation bias, where heavy reliance on external agents can reduce active processing and undermine self-efficacy when overused [3,44].

Although these single items were not included in the core factor model, their inclusion provided valuable contextual information. For instance, the subset of students endorsing “relying on AI makes me doubt my abilities” also tended to score high on redundancy beliefs and low on intrinsic motivation (r ≈.30 with the Redundancy factor). This pattern suggests a potential cycle in which reliance on AI could diminish confidence, which in turn may reduce engagement and promote amotivation—a dynamic consistent with SDT accounts of competence frustration [13]. Conversely, those who felt “more capable with AI” tended to show relatively healthier profiles (e.g., positive association with identified motivation, r ≈.25). This pattern is compatible with the view that when AI is used as a tool for mastery, competence can be bolstered and motivation supported [9,13]. Future research would benefit from expanding these need-focused items into fuller multi-item scales to capture AI-related autonomy and competence satisfaction/frustration with greater reliability and construct coverage [21,40].

In summary, the factor-analytic results indicated that AIM-N captures distinct yet interrelated constructs anchored in SDT: an AI-related meaning dimension (redundancy vs. purpose), an orientation dimension (intrinsic/identified vs. controlled motives), and need-based perceptions (autonomy/competence with AI). These constructs offer a framework for theorizing how AI may alter motivational dynamics in coursework. Notably, identified motivation remained high while intrinsic motivation appeared somewhat dampened. One interpretation is that students continue to perceive value in learning (identified regulation) even when enjoyment or challenge is reduced—possibly because AI removes parts of the task that ordinarily provide satisfying challenge. This nuance has instructional implications: to preserve enjoyment, assignments may need to retain elements of novelty, optimal challenge, and discovery that are not easily offloaded to AI [7,13]. Such design choices are consistent with a need-supportive approach to educational technology integration [9,13].

### Measurement invariance and scale fairness

A critical contribution of the study was the demonstration that the AIM-N scale functions equivalently across key student subgroups. Evidence supported configural, metric, and scalar invariance (or partial scalar with minimal deviations) across gender, study level, field, and AI usage frequency, in line with best practices for invariance testing [23,39,45,46]. Gender invariance indicates that male and female students interpret and respond to AIM-N items similarly; for example, a response of “4” on “AI makes coursework feel like formality” reflects the same latent standing for both genders. This supports meaningful between-group comparisons without measurement bias [47]. In these data, no significant gender differences emerged in the latent factors, suggesting that both men and women included individuals who reacted negatively to AI and individuals who remained self-motivated, contra simple stereotypes about techno-enthusiasm or anxiety.

Study-level invariance (undergraduate vs. graduate) largely held, with one caveat: an item about the degree’s value in an AI future showed different intercepts. This was interpreted as undergraduates being slightly more worried about future degree value than graduate students, even at equivalent levels of redundancy beliefs. Partial scalar invariance appropriately accommodated that difference [23,40]. Aside from this, the scale behaved similarly across educational stages, supporting use in diverse cohorts and suggesting that interventions addressing AI-related demotivation may require tailoring (e.g., reassurance about long-term relevance may be more pertinent for undergraduates).

Field-of-study invariance proved the most complex due to disciplinary diversity and smaller cell sizes. After grouping disciplines, scalar invariance was achieved aside from two items identified as DIF: the “competitive pressure” AI item and the “degree losing value” item, each more strongly endorsed by STEM/business students. These were precisely the direct effects modeled in MIMIC, and the convergence between partial invariance results and MIMIC-DIF strengthens confidence that these differences are specific and substantive [24,41,48]. Importantly, the remaining items—including intrinsic motivation items and most redundancy items—displayed equivalent loadings and intercepts across fields, indicating that AIM-N is suitable for multidisciplinary samples. When raw score comparisons across fields are required, awareness of the two flagged items is advisable; for example, a STEM student may score about 0.4 points higher on “degree losing value” at the same latent redundancy level as a non-STEM peer. Options include contextualizing examples by field, adjusting for field in analyses, or applying MIMIC-based DIF adjustments, depending on the study purpose [24,40].

Finally, invariance across AI usage frequency was examined—an important, often overlooked aspect. Configural and metric invariance were supported, while scalar invariance required partial frees. This pattern is understandable: never-users may respond differently where items are experiential (e.g., “I feel in control when using AI”). Freeing the intercept of such items acknowledged a lower baseline among never-users while preserving the measurement structure. After these adjustments, scalar invariance held, supporting latent mean comparisons between never-users and users. The MIMIC latent means analysis indicated that never-users showed lower redundancy beliefs, consistent with the interpretation that students who have not integrated AI tend not to endorse the idea that AI renders coursework pointless. Thus, AIM-N can be administered regardless of prior AI experience; inclusion of never-users is appropriate so long as interpretation acknowledges systematic differences on a small subset of items.

In psychometric terms, establishing invariance supports the claim that AIM-N is a fair and transportable measure. The scale can be used to track change and compare groups across contexts in science and technology education. When the instrument is deployed in new settings, at least metric invariance should be verified, with attention to the DIF-prone items flagged here. In targeted subpopulations, removing or rewording those items may reduce bias; however, retaining them with analytic adjustments can be informative because they capture substantive field-specific pressures that are part of the AI era in higher education [7,40].

### Drivers of AI-related motivation: What the MIMIC model reveals

One aim of the study was not only to validate the scale but also to identify which students appear more susceptible to AI-related motivational risks. The MIMIC structural analysis yielded several insights consistent with emerging scholarship on generative AI, motivation, and technology-enabled offloading.

#### 1. High AI usage is linked to demotivation and external regulation.

It was found that more frequent AI use in academic work was associated with stronger redundancy beliefs (that tasks feel pointless) and greater endorsement of controlled reasons for engaging in coursework (e.g., deadlines/pressure), with effects of approximately β ≈.30. Although causality cannot be established, the pattern is consistent with research on cognitive offloading and automation bias, which can reduce active processing and promote over-reliance on external tools [3,4,44]. These field-independent mechanisms echo educators’ concerns that heavy reliance on generative AI may erode opportunities to practice skills and dampen autonomous engagement, even when students still recognize the value of learning [7]. The current results also indicated no significant reduction in identified motivation among heavy users; thus, frequent users may still value learning but appear more strategic—using AI to dispose of perceived “busywork” while reporting more external pressure and cynicism about tasks. From the perspective of self-determination theory, such usage patterns may threaten autonomy and competence satisfaction over time, risking drift toward amotivation if offloading becomes habitual [9]. These dynamics emphasise the need for longitudinal designs to test whether early heavy AI use predicts later declines in intrinsic motivation or performance.

Conversely, low or non-use of AI was associated with lower redundancy beliefs and less controlled motivation. This could indicate either that students already high in intrinsic/identified motives were less inclined to adopt AI intensively, or that abstaining/mindful use preserves engagement by maintaining a sense of ownership over effort and outcomes [9]. A plausible moderator concerns how AI is used: augmentation (planning, reviewing, editing) may preserve meaning better than substitution (direct generation), a principle reflected in recent higher-education guidance that positions AI as a complement rather than a replacement for core cognitive activity (e.g., discipline-sensitive recommendations to integrate AI without displacing critical thinking). Future work should therefore disaggregate use-types (generation vs. editing vs. feedback) and map them to motivational outcomes.

#### 2. Field of study plays a significant role in AI–motivation dynamics.

Clear field differences were observed. STEM students, particularly in computing and engineering, exhibited higher redundancy beliefs and more controlled motives. Several mechanisms are plausible. First, many STEM tasks (e.g., code writing) are directly supported by generative AI, making capability substitution salient and thus more likely to trigger redundancy beliefs; empirical work on AI code assistants shows sizeable performance gains on programming tasks, reinforcing the perception that coursework can be expedited or bypassed [49]. Second, competitive, performance-oriented climates—more typical in some technical and business programs—can amplify peer pressure to adopt productivity tools, aligning with the strong “competitive pressure” response pattern in the present data and with literature linking competitive climates to stress and externalized regulation (e.g., person–environment fit work on competitive school climates). Third, pragmatic goal orientations may be more common in some applied fields; when combined with high AI affordances, this orientation can further tilt motivation toward instrumental, outcome-focused engagement.

In contrast, students in Social Sciences, Humanities, and Education showed lower redundancy beliefs and less controlled motivation. This aligns with discipline-sensitive adoption patterns: across institutions, AI engagement is typically higher in engineering/natural sciences and relatively lower in arts/humanities, with differences partly attributable to task types (routine vs. cognitive/interpretive) and disciplinary epistemologies (e.g., “hard/soft” and “pure/applied” distinctions) (Smart Learning Environments synthesis). These differences may also reflect perceived irreplaceability of human-centered, dialogic, and interpretive work (e.g., teaching practice) in such fields, consistent with the lower worry that degrees will “lose value.”

Finally, a STEM–non-STEM contrast emerged for identified motivation: STEM students were slightly less likely to endorse studying “because understanding matters to me.” This pattern is compatible with an outcome-oriented learning stance in some technical programs and raises a specific instructional implication: in fields where AI can readily perform core task components, instructors may need to make the human learning value conspicuous (e.g., debugging heuristics, conceptual modeling, reflective judgment) and to establish norms that reduce arms-race dynamics around tool use. Discipline-specific policies clarifying when not using AI is acceptable (and valued) may also help relieve perceived competitive pressure without disallowing responsible augmentation.

#### 3. Gender and level appear negligible in structural relations.

The absence of gender differences in structural paths is notable. In this sample, male and female students reported comparable AI use and similar motivational impacts, contradicting early conjectures that men (overrepresented in some technology programs) would both adopt AI more readily and experience stronger downstream motivational effects, or that women would be more apprehensive about AI [50,51]. After accounting for field and usage, no direct gender effects were observed. It remains possible that any apparent gender gaps in prior work are mediated by disciplinary composition or usage intensity rather than gender per se [5,52]. From an equity standpoint, this is encouraging: the new motivational challenges posed by AI do not appear to differentially burden one gender in this context. Identified regulation was high and intrinsic motivation moderate for both men and women, consistent with research suggesting that social influence and performance expectancies—rather than gender alone—primarily shape AI adoption and use in higher education [51,53,54].

A similarly small pattern characterized study level. Beyond a modest tendency for postgraduates to report less worry about AI—a developmentally plausible difference given greater academic experience—undergraduates and postgraduates responded similarly. This indicates that AIM–N is appropriate from late undergraduate through graduate studies and that AI-related motivational dynamics are not confined to novice learners. Even doctoral participants (albeit few) endorsed redundancy concerns and AI-related motivations, implying that deliberations about AI’s role in learning extend into advanced study. Comparable undergrad/postgrad patterns have been noted in recent acceptance studies, where performance expectancy dominates for both groups, with other determinants showing only modest between-level variation (Strzelecki, 2025; see also Ravšelj et al., 2025, for mixed socio-demographic effects).

#### 4. Item-level DIF isolates specific, actionable concerns.

The MIMIC analysis highlighted that particular items function differently across groups, highlighting the value of item-level scrutiny for applied use. This is consistent with established guidance that MIMIC models are well suited to detecting uniform DIF with relatively modest sample sizes and clear interpretability of direct effects from grouping variables to indicators [24,41,48,55,56].

One salient example concerned “competitive pressure to use AI.” In contexts with stronger performance/competition climates (e.g., some STEM and business programs), endorsement was elevated, whereas in other fields it was more muted. This pattern coheres with classroom motivation research distinguishing performance-oriented goal structures—where social comparison is salient—from mastery-oriented structures, which emphasize improvement and understanding (Ames, 1992; Midgley et al., 2001/2002). In technology-adoption terms, such local competition likely amplifies social influence pathways central to usage [53]. For AIM–N users, the item functions as a diagnostic: frequent agreement suggests a competitive dynamic around AI that instructors may want to address (e.g., by clarifying AI assessment expectations); low agreement implies competition is not a primary driver in that setting.

A second field-sensitive item concerned “degree losing value.” Technical students agreed more strongly, aligning with broader discourse about automation risk and shifting labor-market skills. Large multi-country student surveys report that learners expect AI to increase demand for AI-related skills and alter job tasks, while expressing ambivalence about the alignment between current education and evolving work requirements [5]. Pedagogically, this item can be read as a barometer of perceived career relevance: concentrations of high scores in specific majors may signal anxiety about credential value. In such cases, explicit attention to durable human capabilities (e.g., judgment, ethical reasoning, collaborative problem solving) and how curricula cultivate them may help sustain motivation without diluting academic standards.

Using DIF findings in practice.

The two flagged items (“stay competitive”, “degree losing value”) should be interpreted with field context in mind. In STEM/business cohorts, elevated endorsement may reflect normative pressures and automation salience, not just individual differences. When these items cluster high, instructors can: (a) explicitly de-emphasize arms-race norms in course policy, (b) build non-automatable components into assessments, and (c) offer career-relevance framing to counter degree-value worries. For cross-group comparisons, analysts may (i) adjust for field, (ii) report latent means rather than raw scores, or (iii) present DIF-aware sensitivity analyses.

### Limitations

Although the study offers several strengths—large sample size, rigorous psychometric procedures, and the integration of multiple theoretical constructs—several limitations should be acknowledged. First, the sample, while large and diverse across fields, was geographically concentrated (primarily Greek university students) so cross-cultural **replication and invariance** tests are warranted. Cultural context may shape how AI is perceived in academic work. In this sample, overall intrinsic motivation remained moderately high; in settings with different educational stakes or technology exposure, responses may differ. Replication in other countries and with younger learners (e.g., high school students) would strengthen generalizability. The survey was administered bilingually and measurement invariance across groups within the sample was established; however, cross-cultural invariance remains an open question.

Second, the data are cross-sectional. C**ross-sectional** data preclude causal inference; longitudinal and experimental designs are needed to test whether AI use **prospectively** raises redundancy beliefs or controlled regulation. Causal directions in the observed associations cannot be confirmed. For instance, higher AI usage was interpreted as leading to higher redundancy beliefs, yet reverse causation is also plausible (i.e., students who already felt less motivated may have turned to AI more frequently). A longitudinal design—measuring motivation before widespread AI use and then after—would help disentangle temporal ordering. The MIMIC model specified covariates (e.g., usage) as influencing latent factors, but experimental or longitudinal evidence is required to substantiate effects of increased AI usage on motivation.

Third, some subscale reliabilities were lower than ideal (particularly for the two-item intrinsic and controlled scales). Lower reliability coefficients for two-item subscales (intrinsic and controlled motivation) are expected given scale length; accordingly, all substantive inferences rely on latent-variable modeling, and future research should expand these dimensions with additional items. T**wo-item** intrinsic and controlled subscales yielded **modest α** (expected with k = 2); all primary inferences therefore rely on **latent-variable models** (ω; CFA) that explicitly account for measurement error, and subscale scores are recommended for **group-level** comparisons rather than individual diagnostics. This pattern is partly attributable to the use of two items; extremely high internal consistency is not expected when only two moderately correlated items are used. This implies that those constructs may be measured with some error. Latent variable modeling was employed to account for measurement error, yet practitioners relying on simple subscale scores should exercise caution. If fine-grained distinctions between intrinsic and controlled motivation are critical, future versions of AIM–N could include additional items to bolster these scales. For example, an item targeting stimulation (another facet of intrinsic motivation, such as excitement when using AI) or an item targeting external rewards (another facet of controlled regulation) may improve reliability. Instrument length was intentionally kept reasonable and key facets were targeted; follow-up work could refine item content.

Relatedly, single-item measures (amotivation, AI-need items) cannot capture the full breadth of those constructs. The **single-item** amotivation and AI-context need items provide **breadth not depth**; future versions should include multi-item banks for AI-autonomy/competence and amotivation. These indicators were treated as standalone and were not relied upon for primary conclusions, but they add context to the overall picture. In future refinements, multi-item subscales for “AI-related amotivation” (e.g., an item such as “I only do schoolwork because I have to, since AI could do it anyway”) or for AI-related autonomy (e.g., additional scenarios reflecting pressure to use AI) would be beneficial. Such items were not included initially to avoid excessive overlap between redundancy beliefs and amotivation, but this remains a promising area for expansion.

Another limitation concerns the rapidly evolving nature of AI tools. Data were collected in mid-2025, and AI capabilities and educational policies are changing quickly. Student attitudes may shift as students and instructors gain more experience with AI. For instance, if instructors integrate AI in meaningful ways, AI may be seen less as undermining learning and more as enhancing it. The present data likely represent an early-phase snapshot (only about 12% identified as “very often” users). As usage becomes more ubiquitous, some relationships may attenuate, while new concerns (e.g., over-reliance leading to skill decay) may emerge. Periodic re-validation of AIM–N is therefore advisable as contexts evolve (the invariance testing across usage groups reported here provides an initial step).

Although the MIMIC framework provides an efficient approach to detecting uniform DIF while preserving model parsimony, it does not test non-uniform DIF (i.e., group differences in factor loadings). To mitigate this limitation, MIMIC analyses were complemented by multi-group CFA invariance testing. Future research could extend these analyses using IRT-based DIF methods or multi-group MIMIC extensions.

Finally, social desirability and response biases could have affected responses. Some students may have under-reported AI usage (given academic honesty concerns) or over-stated positive motivations (e.g., “I value learning”) due to perceived expectations. These risks were mitigated through anonymity and instructions emphasizing honest responses, as well as the inclusion of reverse-worded and negatively framed items to counter acquiescence. The presence of substantial variance and candid endorsements (e.g., agreement with statements questioning the value of doing work if AI can) suggests that many responded openly. Nonetheless, mixed-methods approaches (e.g., interviews) could complement these findings and elucidate the reasoning behind students’ ratings.

### Practical implications

The validated AIM-N scale is a useful tool for educators, counselors, and researchers to diagnose and monitor students’ motivational health in an AI-pervasive learning environment. It can serve multiple practical purposes:

- **Classroom diagnostics:** An instructor could administer a subset of AIM-N items at the start of a course to gauge students’ mindsets. For example, if a significant number of students agree that “I find it hard to stay motivated in courses where AI could do the tasks,” the instructor is alerted that course design might need adjustment (perhaps integrating more authentic tasks or explicitly discussing AI’s appropriate role). If many students indicate they only use AI to meet deadlines, the instructor might incorporate more process-oriented or reflective assignments to cultivate intrinsic interest. By identifying high redundancy belief or low self-determined motivation early, interventions (like a discussion on why human learning still matters, referencing current research or employer expectations) can be targeted.
- **Advising and counseling:** Academic advisors can use AIM-N responses to identify students at risk of disengagement. A student who strongly agrees across redundancy items and shows low intrinsic/identified motivation is one who might be feeling disillusioned about their education (“What’s the point if AI can do it?”). Advisors could explore these feelings in advising sessions, help reframe the student’s goals, or suggest ways to rekindle interest (such as project work emphasizing creativity or human skills). Similarly, knowledge that STEM students, for instance, are more likely to harbor these beliefs means career services in STEM departments might want to proactively address AI anxiety by highlighting continuing value of those degrees or new career paths emerging alongside AI.
- **Curriculum and policy:** At the institutional level, aggregated AIM-N data can inform policy. If across the university many students feel pressure to use AI to keep up, maybe there is a competitive misuse emerging – the institution might then issue guidelines or honor code clarifications on AI use to reduce unhealthy pressure. If large numbers of students in certain majors feel their degree is losing value, perhaps curriculum committees will consider updating content to include more uniquely human skill training or discuss AI openly in classes to validate and relieve these concerns. Over time, tracking AIM-N scores could gauge if interventions (like an AI ethics seminar or integration of AI literacy) are improving student outlook (e.g., do redundancy beliefs go down after we implement an AI across the curriculum program that emphasizes human-AI collaboration?).
- **Research and evaluation:** For researchers, AIM-N opens new avenues to study how technology affects motivation. For example, one could use it to evaluate an experimental condition: if one group of students completes an assignment with AI assistance and another without, how do their AIM-N scores differ afterward? Does using AI on a task increase or decrease their intrinsic motivation for similar tasks? The subscales allow fine-grained analysis – perhaps using AI might lower task-specific intrinsic motivation but not touch identified motivation for the subject as a whole. Without such a scale, these nuanced effects would be hard to quantify. This study provides initial evidence that frequent AI use correlates with certain motivational shifts; experiments can build on this to test causation.

Additionally, the basic needs items indicate areas for direct pedagogical action. For instance, the fact that some students don’t feel in control or feel pressured in using AI suggests instructors should give more autonomy support regarding AI (e.g., allow students choice in how much to use AI, reassure them it’s okay not to use it in certain assignments, thereby reducing felt pressure). The competence-related responses show some students feel more capable with AI – educators can leverage this (AI as scaffolding) but should simultaneously ensure students are also building their own competence (perhaps gradually reducing AI support). Students who doubt their abilities due to AI might benefit from opportunities to perform without AI to rebuild self-efficacy (“Yes, you can write a solid essay even without GPT”).

One surprising positive note was that identified motivation remained high overall. This implies that, despite the disruptive nature of AI, most students still see personal value in learning. Educators should nurture this by explicitly connecting course activities to students’ values and goals. Since that flame is not extinguished, it can be stoked: discussions about academic purpose in the AI era could help students frame why learning is still meaningful (e.g., “Even if AI can do X, understanding X yourself trains your critical thinking for problems Y and Z”). In fact, this instrument’s inclusion of meaning/purpose items (adapted MLQ) – not fully reported here – can further help to integrate those conversations, ensuring that the presence of AI doesn’t inadvertently lead to a nihilistic attitude toward education.

Further action steps for instructors and program leaders would be (i) Assessment redesign to emphasize process, reflection, and authentic tasks that resist trivial automation; (ii) AI-literate autonomy support (clear choices for when/how AI can be used, plus explicit permission *not* to use AI where appropriate) to reduce competitive pressure; (iii) Competence scaffolding with gradual removal of AI supports to rebuild self-efficacy; (iv) Field-sensitive messaging, especially in STEM/business, addressing “degree value” anxieties and highlighting durable human capabilities (judgment, ethics, teamwork, abstraction). Unit-level AIM–N snapshots can guide which lever to prioritize.

### Future work

This research opens several pathways for continued inquiry. Longitudinal designs are needed to examine how students’ motivational responses to AI evolve over time. A cohort could be followed from freshman to senior year to assess whether redundancy beliefs increase as AI tools become more powerful or as students witness additional demonstrations of AI proficiency, or whether students adapt—particularly if curricula are adjusted—such that AI is construed as calculator-like augmentation rather than a replacement for learning. Relatedly, it will be important to test whether intrinsic motivation rebounds after an initial novelty dip once generative AI becomes normalized in coursework. Repeated administrations of AIM-N would allow trajectories to be mapped, provided longitudinal measurement invariance is established so that score changes can be interpreted as true change rather than measurement artifacts [40,57,58].

Intervention studies constitute a natural next step. With a validated measure, randomized and quasi-experimental designs could evaluate strategies intended to mitigate negative motivational effects. For example, brief mindset interventions and relevance-framing activities have shown durable benefits for motivation and achievement in higher education contexts [59,60,61]. Parallel designs could be adapted to the AI context (e.g., workshops emphasizing growth mindset with AI and the value of human–AI collaboration) and compared with business-as-usual controls. If effective, post-intervention AIM-N scores would be expected to show lower redundancy and maintained intrinsic motivation in treatment groups relative to controls—offering evidence-based practices for instructors [7,62].

Future work should also examine individual-difference moderators beyond field and usage. Personality, epistemic beliefs, and achievement goals are likely to shape how AI influences motivation. Students with a strong mastery orientation may persist in learning for its own sake (thus preserving identified motivation), whereas performance/avoidance orientations may increase tendencies to offload effort to AI and risk amotivation [3,63,64]. Prior self-efficacy and achievement may similarly condition responses, with high-efficacy students more likely to appropriate AI as a tool while maintaining engagement [65,66]. Testing these interactions would help identify student profiles most at risk of AI-induced demotivation.

Expanding the instrument to capture collaborative and social dimensions also appears warranted. AI may shift the balance of peer interaction and instructor contact, with implications for relatedness need satisfaction. In the present study, an item was included—“I feel lonely when I am learning” (a relatedness-frustration item); notably, the data showed moderate responses. Future AIM-N versions could incorporate items such as “Using AI instead of asking peers or instructors makes me feel less connected” to index social displacement, social presence, and relatedness—all constructs that have established links to high-quality motivation [11,29,67].

Cross-cultural validation is a critical priority. AIM-N should be translated and validated across languages and educational systems using best practices for test adaptation. Because norms, teaching practices, and attitudes toward technology differ across contexts, measurement invariance should be established across cultural groups before substantive comparisons are made [46]. In settings that emphasize rote learning and high-stakes examinations, redundancy beliefs might be elevated; in inquiry-oriented settings, AI could be positioned as a tool for exploration rather than a substitute for learning. Comparative studies using AIM-N would illuminate such cultural moderators.

Future directions may include (a) Longitudinal models with longitudinal invariance to test change in redundancy beliefs and motivation as AI integration matures; (b) Intervention trials (e.g., relevance-framing, autonomy-supportive AI policies, metacognitive prompts, or phased AI-scaffolding) with AIM–N outcomes; (c) Qualitative studies (interviews/diaries) to unpack when AI sparks curiosity vs. undermines enjoyment; (d) Cross-cultural validation with full multi-group invariance across languages and systems; (e) Use-type taxonomy (augmentation vs. substitution vs. feedback) to explain heterogeneity in motivational effects.

In conclusion, the findings suggest that while AI brings unprecedented capabilities, motivational challenges must be actively managed within courses and programs. The AIM-N instrument provides a means to monitor these challenges and to evaluate the effectiveness of instructional and advising strategies designed to sustain students’ autonomy, competence, and purpose in AI-rich learning environments [9]. As generative AI becomes more deeply integrated into higher education, safeguarding these needs will remain central to ensuring AI functions as a boon rather than a detriment to learning.

## Supporting information

S1 File Supporting Information including raw dataset used for statistical analysis, CodeBook detailing variable definitions and coding schemes and the Research Instrument.
(RAR)
